# Glutathione *S*-Transferase P1 Protects Against Amodiaquine Quinoneimines-Induced Cytotoxicity but Does Not Prevent Activation of Endoplasmic Reticulum Stress in HepG2 Cells

**DOI:** 10.3389/fphar.2018.00388

**Published:** 2018-04-18

**Authors:** Yongjie Zhang, Shalenie P. den Braver-Sewradj, Michiel W. den Braver, Steven Hiemstra, Nico P. E. Vermeulen, Bob van de Water, Jan N. M. Commandeur, J. C. Vos

**Affiliations:** ^1^Division of Molecular Toxicology, Department of Chemistry and Pharmaceutical Sciences, Amsterdam Institute for Molecules, Medicines and Systems, Vrije Universiteit Amsterdam, Amsterdam, Netherlands; ^2^Clinical Pharmacokinetics Research Laboratory, School of Basic Medicine and Clinical Pharmacy, China Pharmaceutical University, Nanjing, China; ^3^Division of Drug Discovery and Safety, Leiden Academic Centre for Drug Research, Leiden University, Leiden, Netherlands

**Keywords:** amodiaquine, quinoneimine, cytotoxicity, endoplasmic reticulum stress, human glutathione *S*-transferases P1

## Abstract

Formation of the reactive amodiaquine quinoneimine (AQ-QI) and *N*-desethylamodiaquine quinoneimine (DEAQ-QI) plays an important role in the toxicity of the anti-malaria drug amodiaquine (AQ). Glutathione conjugation protects against AQ-induced toxicity and GSTP1 is able to conjugate its quinoneimine metabolites AQ-QI and DEA-QI with glutathione. In this study, HepG2 cells transiently transfected with the human *GSTP1* construct were utilized to investigate the protective effect of GSTP1 in a cellular context. HepG2 cells were exposed to synthesized QIs, which bypasses the need for intracellular bioactivation of AQ or DEAQ. Exposure was accompanied by decreased cell viability, increased caspase 3 activity, and decreased intracellular GSH levels. Using high-content imaging-based BAC-GFP reporters, it was shown that AQ-QI and DEAQ-QI specifically activated the endoplasmic reticulum (ER) stress response. In contrast, oxidative stress, DNA damage, or inflammatory stress responses were not activated. Overexpression of GSTP1 resulted in a two-fold increase in GSH-conjugation of the QIs, attenuated QI-induced cytotoxicity especially under GSH-depletion condition, abolished QIs-induced apoptosis but did not significantly inhibit the activation of the ER stress response. In conclusion, these results indicate a protective role of GSTP1 by increasing enzymatic detoxification of AQ-QI and DEAQ-QI and suggest a second protective mechanism by interfering with ER stress induced apoptosis.

## Introduction

**FIGURE 1:**
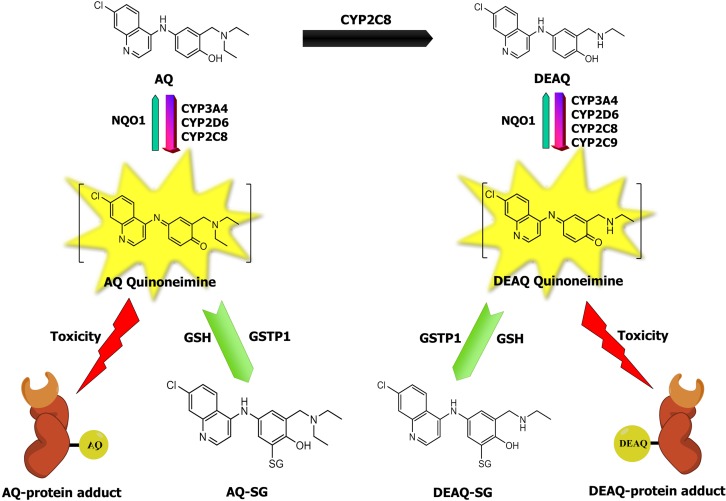
The relationship between AQ metabolism and toxicity. AQ to its major metabolite DEAQ is predominantly catalyzed by CYP2C8 and forms DEAQ (Li et al., 2002). AQ and DEAQ are bioactivated to their corresponding reactive quinoneimine (QI), catalyzed by CYP3A4, CYP2D6, CYP2C8, and CYP2C9 (Zhang et al., 2017b). Protein-reactive QIs of AQ and DEAQ can bind to cellular proteins and subsequently lead to toxicity (Harrison et al., 1992; Naisbitt et al., 1998). Reactive QIs can be detoxified by conjugation reaction with glutathione, which is catalyzed by GSTs, and by reduction reaction catalyzed by human NQO1 (Zhang et al., 2017a).

Amodiaquine is a potent anti-malaria drug, however, rare but life-threatening idiosyncratic hepatotoxicity (1/15,500 patients) and agranulocytosis (1/2000 patients) have restricted its therapeutic use (Olliaro and Mussano, 2003; Walgren et al., 2005). Currently, AQ is forbidden for prophylactic use and is only prescribed in combination therapy with artesunate, as recommended by WHO (2015). AQ undergoes rapid and extensive hepatic metabolism to its pharmacologically active metabolite DEAQ, predominantly by cytochrome P450 2C8 (CYP2C8) (Li et al., 2002). Both AQ and DEAQ are prone to enzymatic oxidation leading to the formation of AQ-QI and DEAQ-QI (Lobach and Uetrecht, 2014; Zhang et al., 2017b). These quinonemines (QIs) can rapidly and irreversibly modify proteins (Maggs et al., 1987; Tingle et al., 1995; Lohmann and Karst, 2007) (**Figure 1**). Such haptenated proteins can disrupt protein folding or function and lead to direct toxicity, or can be recognized as ‘non-self’ leading to immune-mediated toxicity (Cho and Uetrecht, 2017). Congruently, cellular studies demonstrated a significant contribution of AQ bioactivation to AQ cytotoxicity in isolated hepatocytes (Tafazoli and O’Brien, 2009; Peyre et al., 2015) and in neutrophils (Naisbitt et al., 1998). Nevertheless, although it is generally accepted that reactive metabolite formation plays an important role in AQ-induced toxicity (Srivastava et al., 2010; Cho and Uetrecht, 2017), the underlying mechanisms and pathways involved remain unclear. So far, identified contributing factors include oxidative metabolism (either by CYPs or peroxidases/hydrogen peroxide), depletion of cellular GSH, protein carboxylation, mitochondrial disturbances, reactive oxygen species formation, and lipid peroxidation (Naisbitt et al., 1998; Tafazoli and O’Brien, 2009; Heidari et al., 2014; Peyre et al., 2015).

Glutathione *S*-transferases are important enzymes that catalyze the conjugation reaction between GSH and chemically reactive drug metabolites. Furthermore, GSTs are additionally identified as regulators of cellular signaling pathways, thereby influencing cell proliferation or apoptosis (Tew and Townsend, 2012; Board and Menon, 2013). GSH-conjugation by GSTs has been shown *in vitro* with chemically reactive metabolites derived from several drugs displaying idiosyncratic toxicity, including troglitazone, acetaminophen, clozapine, and diclofenac (Dragovic et al., 2010; Okada et al., 2011; Vredenburg et al., 2014; den Braver et al., 2016). We recently demonstrated that GSTs, in particular GSTP1, exhibit high activity in catalyzing the GSH-conjugation of AQ-QI and DEAQ-QI using purified human GSTs (Zhang et al., 2017a). However, whether GSTs can protect against AQ-QI- and DEAQ-QI-induced cytotoxicity has not been evaluated. Nevertheless, several cellular studies have suggested the protective roles of chemical anti-oxidants, such as GSH, as well as drug metabolizing enzymes, such as NQO1 and UDP-glucuronosyltransferases (UGTs), against AQ-induced cytotoxicity (Tafazoli and O’Brien, 2009; Heidari et al., 2014).

HepG2 cells have been used for decades as a test system for studies involving hepatotoxic compounds. However, basal levels of phase I and most phase II drug metabolizing enzymes in HepG2 cells are very low compared to human hepatocytes (Wilkening et al., 2003; Sison-Young et al., 2015). Upon transfection or transduction with genes encoding for one or multiple drug metabolizing enzyme genes, HepG2 cells have been shown to be a valuable model system to study the role of bioactivating enzymes in the cytotoxicity of toxicants (Vignati et al., 2005; Hosomi et al., 2011; Iwamura et al., 2011; Tolosa et al., 2013; Xuan et al., 2016). Thus, in the present study HepG2 cells were utilized in combination with transient transfection of the human *GSTP1* gene.

The aims of the present study are (i) to characterize the mechanisms and cellular pathways of toxicity induced by reactive QIs of AQ; and (ii) to evaluate the ability of GSTP1 in protecting against AQ-QI- and DEAQ-QI-induced cytotoxicity. To this end, we evaluated multiple cellular parameters including loss of cell viability, caspase 3 activation, GSH-conjugate formation, GSH homeostasis, and cellular stress response pathway activation in mock- and *GSTP1*-transfected HepG2 cells. Particularly, the activation of adaptive stress response pathways by AQ-QI and DEAQ-QI was elucidated using high-content imaging-based BAC-GFP toxicity assays. To circumvent the need for intracellular bioactivation of AQ or DEAQ by CYPs, the toxic effects of the synthetic chemically reactive metabolites AQ-QI and DEAQ-QI were studied. This study identified ER as the major target for QIs-induced perturbations and a significant protective role of GSTP1 by alleviating loss of cell viability, enhancing detoxification of reactive QIs, and attenuating apoptosis. We propose a model to illustrate the bifunctional potential of GSTP1 in protecting cells from chemically reactive metabolites targeting the ER.

## Materials and Methods

### Caution

The quinoneimines are potential highly-toxic and protein-reactive compounds. Handling requires suitable personal protections such as gloves and safety glasses.

### Chemicals and Reagents

Amodiaquine dihydrochloride was obtained from ICN Biomedicals (Aurora, OH, United States), DEAQ was obtained from BD Biosciences (Franklin Lakes, NJ, United States). BSO, resazurin, and CDNB were purchased from Sigma-Aldrich (Zwijndrecht, Netherlands). AQ-SG) and DEAQ-SG references were synthesized and quantified as described before (Zhang et al., 2017b). Recombinant GSTP1-1 reference was obtained from earlier study in our group (Zhang et al., 2017a).

HepG2 cells were obtained from European Collection of Cell Cultures (ECACC, Salisbury, United Kingdom). DMEM and PBS were purchased from Lonza (Switzerland). FBS was purchased from PAA Laboratories (Austria). GenJet In Vitro DNA Transfection Reagent and buffer were purchased from Tebu-bio (Heerhugowaard, Netherlands). Caspase 3 substrate Ac-DEVD-AMC was purchased from Enzo Life Sciences (Brussels, Belgium).

### Synthesis of Amodiaquine Quinoneimine (AQ-QI) and Desethylamodiaquine Quinoneimine (DEAQ-QI)

Synthesis of AQ-QI and DEAQ-QI was adapted from a method published previously, with a few modifications (Harrison et al., 1992). Briefly, silver oxide was prepared by mixing one equivalent of silver nitrate and 2.5 equivalents of sodium hydroxide in aqueous solution and the solution was stirred on ice for 15 min. The formed silver oxide precipitate was filtered and washed with acetone under vacuum and the resulting dried silver oxide was ready for the subsequent QI synthesis. An excessive (five equivalents) amount of freshly prepared silver oxide was added to one equivalent of AQ or DEAQ in anhydrous chloroform and the mixture was stirred under nitrogen protection for 1 h at room temperature. The formed QIs of AQ and DEAQ were concentrated *in vacuo* and then applied to a silica-60 column to remove the tracing AQ or DEAQ. Identity of synthetic AQ-QI and DEAQ-QI was verified by mass spectrometry and the purities were above 95% (Supplementary Figure S1), as determined by HPLC-UV and LC-TOF-MS (Zhang et al., 2017b). AQ-QI and DEAQ-QI were dissolved in DMF, stored at -80°C and protected from light to prevent possible degradation.

### Cell Culture

HepG2 cells were cultured in collagen-coated plates and maintained in DMEM containing 10% FBS, 1% penicillin/streptomycin (PAA Laboratories, Austria), 1% ultraglutamine (Lonza, Switzerland) and 1% non-essential amino acids (Sigma-Aldrich, Germany). Cells were incubated at 37°C in 5% CO_2_ and 95% humidity and were used up to passage 25. Cells were passaged upon reaching 80% confluency using Trypsin-EDTA (Lonza, Switzerland).

### Transient Transfection of Human *GSTP1* Gene

After plating on collagen-coated plates for 24 h, HepG2 cells were transiently transfected with 0.1 μg/1 × 10^4^ cells *hGSTP1* expression plasmid (SC119655, Origene, Rockville, MD, United States) or accompanying empty pCMV6-XL5 vector (pCMV6-XL5) using the GenJet In Vitro Transfection Reagent for HepG2 cells (SignaGen, Rockville, MD, United States) according to the manufacturer’s instructions. At 18 h after transfection, medium was replaced and cells were cultured for an additional 30 h prior to incubations.

### GSTP1 Activity Assay

HepG2 cells were plated on collagen-coated 6-well plates at 3 × 10^5^ cells per well and transfected as described in the above section. At 48 h post-transfection, cells were harvested in ice-cold PBS using Trypsin-EDTA (Lonza, Switzerland), centrifuged at 1000 × *g* for 3 min, and washed with ice-cold PBS. Cell pellets were re-suspended in 100 μL PBS. Suspended cells were lysed with three freezing-thaw cycles in liquid nitrogen and subsequent ultra-sonication. Cell lysates were obtained with centrifugation at 14000 rpm for 75 min. GSTP1 activity was measured in the supernatant using CDNB as a substrate according to the method described by Habig et al. (1974). GST concentrations in HepG2 cell lysate were estimated based on the specific activity of recombinant GSTP1-1 references. Protein concentrations were determined using the bicinchoninic acid method with bovine serum albumin as standard (Thermo Fisher Scientific, Waltham, MA, United States). Activity assay was carried out after each *GSTP1* transfection as validation for the transfection efficiency.

### Cell Viability Assay

HepG2 cells were plated on collagen-coated 96-well plates at the density of 1 × 10^4^ per well in 0.1 mL DMEM. When applicable, cells were transfected 24 h later as described above. At 48 h after seeding cells were treated without or with 500 μM BSO for 24 h, which reduced GSH levels by more than 90% (data not shown). At 72 h after seeding medium was replaced for DMEM without FBS containing test compound. The percentage of organic solvent (DMF) was kept constant at 0.5%. This DMF concentration didn’t reduce cell viability under non-BSO pretreatment condition and reduced cell viability less than 5% under BSO pretreatment condition (Supplementary Figure S2). Cells were incubated with QIs or parent compounds for 2.5 h, based on the time-dependency of QIs-induced loss of cell viability (Supplementary Figure S3). Following treatment medium was replaced and cell viability was assessed after 16 h (Naisbitt et al., 1998) by the resazurin reduction assay as described before (Sison-Young et al., 2017).

### Determination of Caspase 3 Activity

Cells were plated on black, clear-bottomed collagen-coated 96-well plates at the density of 2 × 10^4^ per well in 0.1 mL DMEM. At 24 and 48 h after seeding, cells were respectively transfected and treated without or with 500 μM BSO as described above. At 48 h following transfection HepG2 cells were treated with 0.1 mL DMEM without FBS containing 50 μM AQ-QI or 30 μM DEAQ-QI for 16 h. Final concentrations of DMF were 0.5%. As a positive control for apoptosis, tamoxifen (50 μM) was included (Mandlekar and Kong, 2001). Following incubation, caspase 3 activity was measured using the fluorogenic substrate AC-DEVD-AMC as described before (Carrasco et al., 2003). Briefly, 50 μL of caspase assay mixture containing 150 mM HEPES, pH 7.4, 450 mM NaCl, 150 mM KCl, 30 mM MgCl_2_, 1.2 mM EGTA, 1.5% Nonidet P40, 0.3% CHAPS, 30% sucrose, 150 μM caspase 3 substrate DEVD-AMC, 30 mM DTT, and 3.0 mM PMSF was directly added to HepG2 cells. Plates were incubated at 37°C for 2 h and then fluorescence was measured with excitation at 360 nm and emission at 460 nm using a ClarioStar Monochromator Microplate Reader (BMG LABTECH, Ortenberg, Germany). Raw fluorescence values were corrected for background fluorescence, which was measured in wells without cells containing only medium, one-step assay buffer and test compound.

### Metabolism of AQ-QI and DEAQ-QI in HepG2 Cells

HepG2 cells were plated on collagen-coated 24-well plates at the density of 6 × 10^4^ per well in 1 mL DMEM. Cells were transfected at 24 h after seeding and subsequently treated without or with BSO at 24 h after transfection, as described above. At 24 h after BSO treatment, HepG2 cells were exposed to 50 μM AQ-QI or 30 μM DEAQ-QI in DMEM medium without FBS for 2.5 h. Incubations were terminated by addition of ice-cold perchloric acid at a final concentration of 1% and plates were kept on ice for 10 min. Cell lysates were transferred to Eppendorf tubes and debris was removed by centrifugation at 14000 rpm for 20 min at 4°C. Supernatants were filtered over 0.2 μm filters and stored at -20°C until analysis. GSH-conjugates of AQ-QI and DEAQ-QI were analyzed and quantified by HPLC-UV, as described previously (Zhang et al., 2017b).

### Assessment of Cellular GSH and GSSG Content

To investigate the GSH and GSSG content in *GSTP1*- and mock-transfected HepG2 cells, concentrations of total GSH and GSSG were measured using the GSH/GSSG-Glo^TM^ Glutathione Assay kit (Promega, Madison, WI, United States). Cells were plated in collagen-coated 96-well plates at the density of 2 × 10^4^ per well in 0.1 mL DMEM. Transfection, BSO treatment, and exposure of AQ-QI and DEAQ-QI were identical as described in section “Transient Transfection of Human *GSTP1* Gene” and “Cell Viability Assay.” After exposure the medium was gently removed and the later steps were conducted according to manufacturer’s manual.

### Stress Reporter Assay of AQ-QI and DEAQ-QI

To identify the stress response pathways upon AQ-QI and DEAQ-QI exposure, oxidative stress response (SRXN1-GFP), ER stress response (CHOP-GFP), inflammation response (ICAM1-GFP), and DNA damage response (p21-GFP) pathways were measured with a high-content image-based BAC-GFP stress responses reporter assay (Wink et al., 2014, 2017).

HepG2-based reporter cells were generated by Bacterial Artificial Chromosome tagging as described previously (Poser et al., 2008) and have been characterized previously (Wink et al., 2017). The modified HepG2 cells originated from HepG2 wild-type cells acquired from ATCC (clone HB8065). The cells were plated on collagen-coated 96-well plates at the density of 2 × 10^4^ per well in 0.1 mL DMEM, treated subsequently as described above.

Levels of GFP-tagged stress proteins were monitored using a Nikon TiE2000 confocal laser microscope (lasers: 488 and 408 nm), equipped with a perfect focus system, a controlled temperature (37°C) and CO_2_ (5%) and an automated stage. Prior to imaging, HepG2 cells were stained with 100 ng/mL Hoechst_33342_ to visualize the nuclei. After 45 min of Hoechst_33342_ staining, medium was replaced with medium containing the different concentrations of AQ-QI, DEAQ-QI, AQ, and DEAQ and monitored for 24 h. Quantification of GFP-tagged stress proteins and data handling were conducted as previously described (Wink et al., 2017).

### Statistical Analysis

Statistical analysis was conducted using Prism (version 5.0). Data were considered significantly different between groups when *p* < 0.05, which was calculated using the Student’s *t*-test (unpaired samples, two-tailed, unequal variance).

For the statistics of stress reporter assay data, R (3.3.2) (R Core Team, 2017) and nlme (Pinheiro et al., 2009) (nlme: Linear and Nonlinear Mixed Effects Model) was used to perform a linear mixed effect model for longitudinal data with the following call: lme [value ∼ time ^∗^ treatment_dose, random = ∼time | plateID, correlation = corAR1 (form = ∼1 |plateID)]. As fixed effects we added time and treatment-dose effects with interaction. As random effects, we had intercepts for plateID (replicates), as well as by-plateID random slopes for the effect of time. Correlation analysis was performed with corAR1, which describes the within group correlation structure as an autoregressive process of order 1, and accounts for correlation between adjoining observations.

Each treatment was compared to the vehicle (DMF) control treatment in either mock or *GSTP1* cells. *p*-values below 0.05 (^∗^), 0.01 (#), and 0.001 (¶) were considered as significant.

## Results

### Validation of Transfection of GSTP1 in HepG2 Cells

To evaluate the role of GSTP1 in protecting against QIs-induced cellular perturbations, HepG2 cells were transiently transfected with a DNA construct containing the human *GSTP1* cDNA behind a CMV-promoter. GST activities in mock- and *GSTP1*-transfected HepG2 cells were characterized by measuring CDNB conjugation to GSH in total cell lysates (**Table 1**). A 29-fold increase in CDNB conjugation was observed in *GSTP1*-transfected cells. GSH depletion in transfected HepG2 cells by BSO-treatment slightly decreased GSTP1 activity in cell lysates. By using the specific activity of purified recombinant GSTP1, the GSTP1 protein concentration in transfected cells was estimated at 40–51 μg/mg cytosolic protein. As GSTP1 appears not to be expressed in HepG2 cells (Sison-Young et al., 2015), the low activity of CDNB conjugation in lysates from mock-transected cells is most likely due to other GST isoforms (Hao et al., 1994; Scharmach et al., 2009).

**Table 1:** Specific activity of GST in HepG2 cells transfected with empty vector and human *GSTP1* gene.

	Mock, No BSO	GSTP1, No BSO	Mock, BSO	GSTP1, BSO
Activity (nmol/min/mg cytosolic protein)	14 ± 2	400 ± 13	19 ± 5	317 ± 15

### Protection by GSTP1 Against AQ-QI and DEAQ-QI Cytotoxicity

**FIGURE 2:**
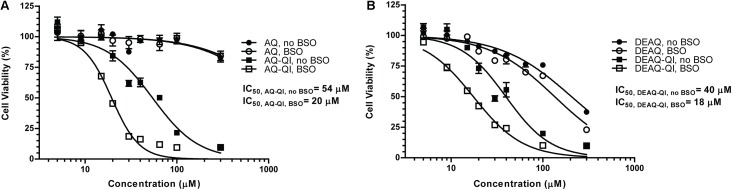
Fitted dose–response curves of viability of HepG2 cells under treatment of AQ or AQ-QI **(A)** and DEAQ or DEAQ-QI **(B)**. HepG2 cells were exposed to test compounds for 2.5 h. GSH depletion was performed by addition of BSO (500 μM) 24 h before the exposure. Cells were recovered overnight and cell viability was measured by resazurin reduction activity. Results are presented as percentage relative to vehicle controls in respective treatment groups. Each data point represents the mean ± SD (*n* = 3).

To investigate the protective effects of GSTP1 under normal and GSH-depleted conditions, BSO was used to disrupt GSH synthesis. BSO treatment alone did not significantly decrease cell viability (data not shown) and had no profound effect on the cytotoxicity of AQ or DEAQ (**Figure 2**). At 300 μM, AQ and DEAQ decreased cell viability with approximately 15 and 70%.

Cytotoxicity of AQ-QI and DEAQ-QI was time-dependent during the first 3 h of exposure (Supplementary Figure S3). Longer incubation times did not increase cytotoxicity. In subsequent experiments, cells were therefore exposed to the synthetic QIs for 2.5 h. AQ-QI and DEAQ-QI showed a comparable dose–response (**Figure 2**). GSH depletion lowered IC_50_ values for both AQ-QI (from 54 to 20 μM) and DEAQ-QI (from 40 to 18 μM).

**FIGURE 3:**
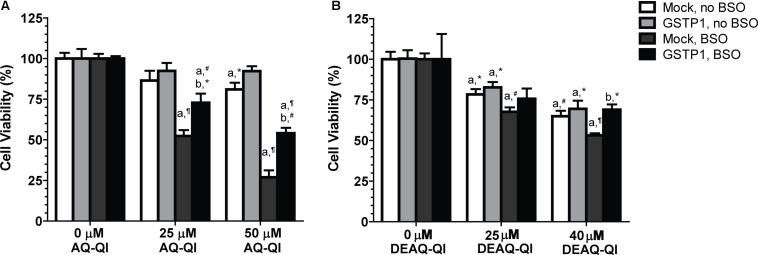
Effect of GSTP1 in protection against AQ-QI- **(A)** and DEAQ-QI- **(B)** induced loss of cell viability. AQ-QI and DEAQ-QI were incubated with 1 × 10^4^ HepG2 cells transiently transfected with mock or *GSTP1* gene for 2.5 h. GSH depletion was performed by addition of BSO (500 μM) 24 h before the exposure of AQ-QI and DEAQ-QI. Cells were recovered overnight and cell viability was measured by resazurin reduction activity. Results were normalized with setting vehicle controls under each treatment at 100%. Results are presented as mean ± SD (*n* = 3). Statistical significant differences with corresponding vehicle control are denoted with ‘a’ and with corresponding mock-transfected control are denoted with ‘b’ using the following *p*-values: ^∗^*p* < 0.05, ^#^*p* < 0.01, ¶*p* < 0.001; analyzed by Student’s *t*-test.

To investigate the cytoprotective role of GSTP1, mock- and *GSTP1*-transfected cells were exposed to AQ-QI and DEAQ-QI. As shown in **Figure 3A**, under GSH-depleted conditions viability of *GSTP1*-transfected cells was significantly higher compared to that of mock cells after treatment with 25 and 50 μM AQ-QI or 40 μM DEAQ-QI. Without GSH depletion, viability of mock- and *GSTP1*-transfected cells was comparable for all treatments.

### Protection Against AQ-QI- and DEAQ-QI-Induced Apoptosis by GSTP1

**FIGURE 4:**
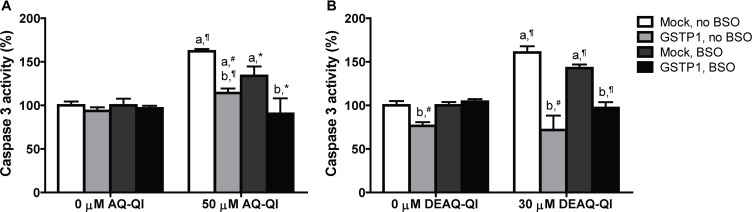
Caspase 3 activity in mock- and *GSTP1*-transfected HepG2 cells following a 2.5 h exposure to 50 μM AQ-QI **(A)** or 30 μM DEAQ-QI **(B)** with or without a 24 h pretreatment with 500 μM BSO. Caspase 3 activity was used as measure for cell death by apoptosis and is presented as percentage relative to respective vehicle controls in mock-transfected cells. Each bar represents the mean ± SD from three independent experiments. Statistical significant differences with corresponding vehicle control are denoted with ‘a’ and with corresponding mock-transfected control are denoted with ‘b’ using the following *p*-values: ^∗^*p* < 0.05; ^#^*p* < 0.01; ¶*p* < 0.001 (Student’s *t*-test).

To test whether AQ-QI and DEAQ-QI exposure induced apoptosis and to study the protective potential of GSTP1, activity of caspase 3 was measured under normal and GSH-depleted conditions (**Figure 4**). GSH depletion by BSO treatment did not significantly increase caspase 3 activity (data not shown). Without GSH depletion, exposure to both AQ-QI and DEAQ-QI increased caspase 3 activity by 60%. When intracellular GSH levels were depleted activation of caspase 3 was less, being 30 and 40% for AQ-QI and DEAQ-QI, respectively. Expression of GSTP1 prevented QI-induced activation of caspase 3 almost completely. Of note is that GSTP1 also attenuated caspase 3 activity induced by the positive control tamoxifen by 50% (Supplementary Figure S4).

### GSTP1 Activity in GSH-Conjugation of AQ-QI and DEAQ-QI

**FIGURE 5:**
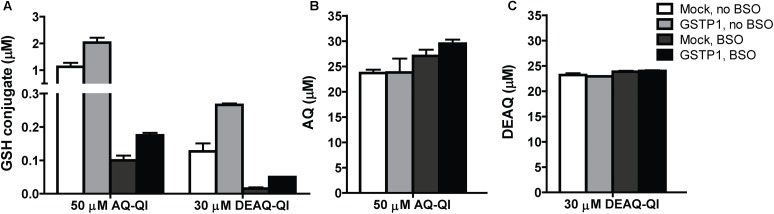
Concentrations of GSH-conjugates of AQ-QI (AQ-SG) and DEAQ-QI (DEAQ-SG) **(A)**, AQ **(B)**, and DEAQ **(C)** in total lysates of mock- and GSTP1-transfected HepG2 cells. When applicable, pre-exposure of 500 μM BSO was performed 24 h before treatment. Cells were treated with 50 μM AQ-QI or 30 μM DEAQ-QI for 2.5 h, after which GSH conjugates and reduces QIs were analyzed using HPLC. Results are presented as mean ± SD from four independent experiments.

To investigate the intracellular GSTP1-mediated GSH-conjugation of AQ-QI or DEAQ-QI, AQ-SG, and DEAQ-SG formation were measured in total lysates directly after QI exposure. Under non-stressed GSH concentrations, GSH-conjugation of AQ-QI and DEAQ-QI was twofold higher in *GSTP1*-transfected cells (**Figure 5A**). Under GSH-depleted conditions, GSH-conjugation was two- and three-fold higher in *GSTP1*-transfected cells compared to mock-transfected cells, for AQ-QI and DEAQ-QI respectively. The concentration of GSH-conjugates was 0.2 to 4% of the initial AQ-QI concentration and 0.05 to 0.9% of the DEAQ-QI concentration (**Figure 5A**). The majority of QIs was reduced back to the respective parent compound (∼50% of AQ-QI and ∼80% of DEAQ-QI), presumably by the action of endogenous antioxidants and/or reductases, e.g., quinone reductases (**Figure 5B**).

### The Effect of GSTP1 on AQ-QI- and DEAQ-QI-Induced Alteration on Cellular GSH Homeostasis

**FIGURE 6:**
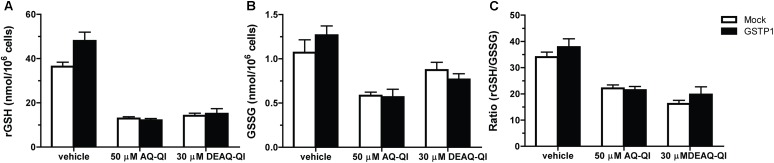
Effects of 50 μM AQ-QI and 30 μM DEAQ-QI on intracellular concentrations of reduced GSH (rGSH) **(A)**, GSSG **(B)**, and on the ratio of reduced GSH to GSSG **(C)** in HepG2 cells without BSO pretreatment. Mock- and *GSTP1*-transfected cells were treated with 50 μM AQ-QI or 30 μM DEAQ-QI for 2.5 h, after which GSH and GSSG levels were quantified as described section “Materials and Methods.” Results are presented as mean ± SD (*n* = 3).

To evaluate whether cellular GSH homeostasis was affected by QIs, and whether GSTP1 did influence the effects, concentrations of intracellular GSH and GSSG were quantified following exposure to synthetic AQ-QI and DEAQ-QI. BSO treatment decreased intracellular GSH levels from 40 to 0.8 nmol/10^6^ cells, and further decreased below detection limits following QI exposure (data not shown). Oxidized GSH (GSSG) levels were not detectable under GSH-depleted conditions. As shown in **Figure 6A**, GSH levels decreased to 25–40% of control cells after AQ-QI or DEAQ-QI treatment, while the intracellular GSSG concentrations did not increase correspondingly, but decreased to approximately 45–80% (**Figure 6B**). Following AQ-QI or DEAQ-QI treatment, the ratio of reduced GSH to oxidized GSH decreased accordingly to 40–65% of control cells (**Figure 6C**). Compared with mock cells, no apparent differences in concentrations of reduced or oxidized GSH were observed in *GSTP1*-transfected cells.

### Activation of Adaptive Stress Responses by AQ-QI and DEAQ-QI and Effects of GSTP1

**FIGURE 7:**
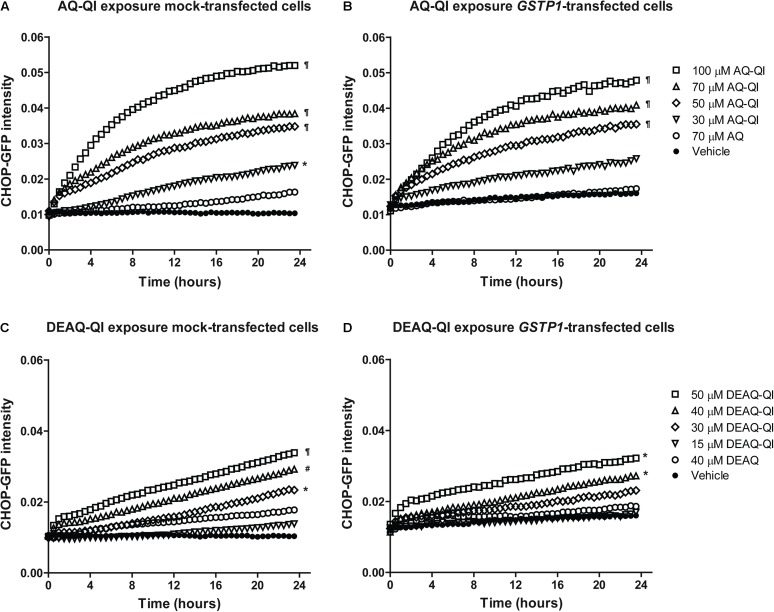
Time dynamics of CHOP-GFP reporter activation following exposure to AQ-QI **(A,B)** or DEAQ-QI **(C,D)**. Prior to exposure, HepG2 DDIT3/CHOP-GFP cells were transfected with empty vector **(A,C)** or *GSTP1* cDNA **(B,D)**. Statistical significant differences with corresponding vehicle control from each group are denoted with ^∗^*p* < 0.05, ^#^*p* < 0.01, and ¶*p* < 0.001; analyzed by linear mixed effect model as described in detail in section “Materials and Methods.”

The stress responses induced by AQ-QI and DEAQ-QI exposure were measured with a high-content image-based BAC-GFP stress responses reporter assay for oxidative stress, ER stress, DNA damage stress and inflammatory stress (Wink et al., 2014, 2017). BSO treatment was not included, because this already induced a significant oxidative stress response (data no shown). Up to respectively 70 and 40 μM, AQ or DEAQ did not significantly activate any of the tested stress response pathways, which is consistent with the limited onset of cytotoxicity by these compounds (**Figure 2**). However, exposure to AQ-QI or DEAQ-QI resulted in a rapid, marked activation of the ER stress response. Interestingly, all other tested pathways (oxidative stress, DNA damage, and inflammatory stress) were not significantly activated by either AQ-QI or DEAQ-QI treatment (Supplementary Figure S5). As shown in **Figure 7**, in mock- and *GSTP1*-transfected HepG2 cells dose- and time- dependent up-regulations of CHOP-GFP signal were detected following treatment with AQ-QI or DEAQ-QI. Although a minor inhibition of the ER stress response by GSTP1 was observed (comparing corresponding curves in **Figures 7A–D**), no statistical difference was found between mock- and *GSTP1*-transfected cells.

## Discussion

The contribution of oxidative metabolism of AQ to cytotoxicity has previously been demonstrated by several studies (Naisbitt et al., 1998; Tafazoli and O’Brien, 2009; Peyre et al., 2015). Recently, it was shown that recombinant human GSTs, in particular GSTP1, significantly contributed to the inactivation of AQ-derived QIs by catalyzing GSH-conjugation (Zhang et al., 2017a) (**Figure 1**). GSTP1 is generally not expressed in human hepatocytes (Sison-Young et al., 2015), however, expression is often up-regulated under stress conditions and in tumors (Tew and Townsend, 2012). GSTP1 is highly expressed in neutrophils (Fessler et al., 2002), where it may protect against AQ-induced agranulocytosis. In the present study, GSTP1 was selected as a model GST to evaluate the protective effects of GSTs in a cell-based *in vitro* system.

Glutathione *S*-transferase activities in mock- and *GSTP1*-transfected HepG2 cells were in a similar range as reported previously in mock- (and *GSTP1*-)transfected HepG2 cells (Hu et al., 1999; Gallagher et al., 2007; Peklak-Scott et al., 2008). Low basal level of CDNB activity observed in mock HepG2 cells likely resulted from low levels of other GST isoforms, since GSTP1 is not expressed in HepG2 cells (Sison-Young et al., 2015). The 29-fold increase of CDNB activity observed in *GSTP1*-transfected cells unambiguously demonstrated the over-expression of functional GSTP1.

We circumvented the need for CYP-mediated bioactivation of AQ and DEAQ by exposing the cells to the synthetic AQ-QI and DEAQ-QI, the reactive metabolites of AQ. As expected, the chemically reactive metabolites were much more cytotoxic than their respective precursors and displayed comparable and steep dose-dependent decreases in cell viability (**Figure 2**). Cytotoxicity of both QIs increased under GSH-depleted conditions, confirming that GSH is important as scavenger by GSH-conjugation and as endogenous antioxidant (**Figure 1**). In contrast, caspase 3 activation, a marker for apoptotic activation, by AQ-QI and DEAQ-QI was higher without BSO pre-treatment (**Figure 4**). The difference between the effects of both QIs on the cell viability (**Figure 2**) and caspase 3 activation (**Figure 4**) suggests that upon GSH depletion, the decrease in cell viability is not only apoptosis dependent but also most likely depends other cell death pathways, such as necrosis (Iorga et al., 2017).

IC_50_ values of QIs under GSH-depleted conditions were around 20 μM (**Figure 2**), which is higher than the plasma concentrations of AQ and DEAQ found in patients, normally in nanomolar to micromolar ranges (Laurent et al., 1993; Lai et al., 2009; Scarsi et al., 2014). However, hepatic concentrations of AQ and DEAQ are likely higher as AQ strongly accumulates in the liver (Barrow, 1974). Although QI levels *in vivo* may be lower, exposure continues for a prolonged period as the half-life of DEAQ is 10 to 12 days (Krishna and White, 1996; Rijken et al., 2011). In the current HepG2 cell model, in which prolonged exposure is not feasible, the use of relatively high QI concentrations in a short timeframe allows for the identification of underlying mechanisms and pathways, as has been done previously in similar studies with hepatic toxicants (Tafazoli and O’Brien, 2009; Heidari et al., 2014) and other reactive drug metabolites such as NAPQI (Chia et al., 2010; Kalinec et al., 2014).

The relative contribution of GSTP1 to GSH-conjugation of both QIs was highest at GSH-depleted conditions, while in absolute quantities a higher GSTP1-dependent increase of GSH conjugate formation was seen without BSO pre-treatment (**Figure 5**). Additionally, *GSTP1*-transfected HepG2 cells were significantly more resistant against the cytotoxicity of both QIs under GSH-depleted conditions (**Figure 3**). Together, these results indicate a more critical role of GSTP1 under low GSH concentration. The more efficient conjugation of the QIs to GSH may have reduced covalent modification of cellular macromolecules, like ER proteins Grp78/Bip (Xu et al., 2015), microsomal GST (Weis et al., 1992), and PDI (Liu et al., 2005). GSTP1 itself can also function as a target, as has been shown for chemically reactive metabolites derived from acetaminophen (Jenkins et al., 2008) and diclofenac (Boerma et al., 2012).

Although reactive QI formation has been associated with AQ-induced (cyto)toxicity *in vitro* (Naisbitt et al., 1998; Tafazoli and O’Brien, 2009) and *in vivo* (Christie et al., 1989; Shimizu et al., 2009), the exact cellular mechanisms and pathways of AQ-QI and DEAQ-QI toxicity remain poorly understood. For GSTs, the protective role toward cytotoxicity or mutagenicity has been shown for many xenobiotics, including environmental carcinogens, such as polycyclic aromatic hydrocarbons (Hu et al., 1999; Ahmad et al., 2008; Kabler et al., 2009), anti-tumor alkylating agents, such as doxorubicin (O’Brien et al., 2000; Tashiro et al., 2001) and cisplatin (Peklak-Scott et al., 2008), and hepatotoxic drugs such as diclofenac (Al-Attrache et al., 2016), isoniazid and acetaminophen (Tolosa et al., 2017). However, also for GSTs, the mechanisms of protection are not always clear. More recently, it is becoming well-recognized that a better understanding of cellular signaling pathways alternation under toxicological insult will benefit the risk assessment and early stage prediction of drug toxicity (Jennings et al., 2013). For this purpose, activation of adaptive stress responses was investigated for DNA damage (p21), oxidative stress (SRXN1), inflammation (ICAMI), and ER stress (CHOP) using a HepG2-based GFP-reporter assay (Wink et al., 2014, 2017). Unexpectedly, activation of p21, ICAM1, or SRXN1 was not observed (Supplementary Figure S5), although DNA (Dybing et al., 1984), TRPA1 (Nassini et al., 2010), and Keap1 (Copple et al., 2008) are known targets for other QIs. The lack of a DNA damage response may be a result of the extracellular exposure to QIs. The modest decrease in GSH/GSSG ratios seen (**Figure 6**) is consistent with the lack of activation of an adaptive oxidative stress response pathway observed here and in a previous study (Tafazoli and O’Brien, 2009). As shown in **Figure 7**, AQ-QI and DEAQ-QI exposure resulted in activation of the ER stress response. Many chemicals have been reported to induce ER stress and subsequently cause toxicity in hepatic and extra-hepatic cells (Fredriksson et al., 2014; Foufelle and Fromenty, 2016; Ren et al., 2016; Qaisiya et al., 2017), in particular chemically reactive benzoquinones which may react directly with ER proteins (Wang et al., 2006; Kalinec et al., 2014). Mild ER stress assists cells with surviving from intrinsic or external insults. However, in case of persistent and/or larger ER stress prevailing over the cellular defensive mechanisms, cell injury and activation of various pro-apoptotic signaling pathways take place (Chaudhari et al., 2014) in which CHOP exhibits an essential role (Oyadomari and Mori, 2004; Tabas and Ron, 2011). The rapid CHOP activation indicated that cytotoxicity of AQ-QI and DEAQ-QI was accompanied by the activation of the adaptive ER stress response pathway and subsequent activation of apoptotic programs (Fredriksson et al., 2014). The initial insult inflicted by the QIs, activating CHOP, could not be lowered by GSTP1. These results indicate that GSTP1 interfered with QI-induced apoptosis downstream of CHOP activation, or that apoptosis was additionally activated via a CHOP independent pathway (Hetz, 2012) with a higher threshold. GSTP1 as a modulator in cell survival and/or apoptotic signaling pathways has been well-established (Tew and Townsend, 2012; Board and Menon, 2013). Drug resistance observed in tumor tissues have been associated with the overexpression of GSTP1, which effectively blocks JNK signaling pathways (Adler et al., 1999) while specific inhibitors of GSTP1 induce activation of JNK (Burg et al., 2006). In the present study, GSTP1-mediated JNK modulation leading to inhibition of pro-apoptotic signaling pathway may have contributed to the protective effect of GSTP1 against QIs-induced cytotoxicity. In line with this hypothesis, activation of caspase 3 by tamoxifen, which is known to cause JNK-mediated apoptosis (Mandlekar and Kong, 2001), was also significantly attenuated by GSTP1 (Supplementary Figure S4), which cannot be explained by GSTP1-mediated inactivation of reactive metabolites in the absence of bioactivation of tamoxifen in HepG2 cells.

**FIGURE 8:**
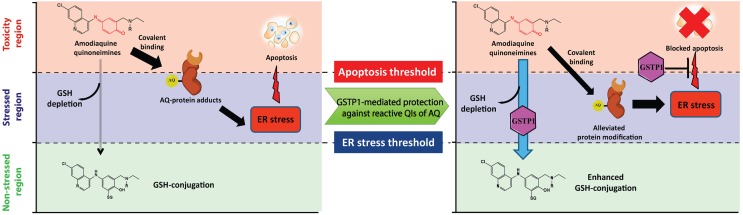
Proposed model for the toxicity of AQ-QI and DEAQ-QI and GSTP1-mediated protection in HepG2 cells. Reactive moieties in the QIs are indicated in red. For AQ-QI, R = -CH_2_CH_3_; for DEAQ-QI, R = -H. AQ-QI and DEAQ-QI deplete cellular GSH and are conjugated into AQ-SG and DEAQ-SG, respectively. The ER stress response (CHOP) is activated by QI-mediated protein modification and apoptosis is subsequently induced. GSTP1-mediated protection against reactive QIs of AQ is due to enhanced GSH-conjugation and inhibition of apoptosis.

In summary, we identified ER stress as the main adaptive stress response caused by AQ-QI and DEAQ-QI and evaluated the protective role of GSTP1 using a transfected HepG2 cell model. Expression of GSTP1 blocked caspase 3 activation and increased GSH-conjugation upon AQ-QI and DEAQ-QI treatment. Furthermore, AQ-QI- and DEAQ-QI-induced cytotoxicity was reduced in *GSTP1*-transfected cells under GSH-depleted conditions. The immediate activation of the adaptive ER stress response was reduced, but not blocked by GSTP1. Altogether, as proposed in the model in **Figure 8**, we conclude that the protein-reactive QIs of AQ mainly trigger ER stress, eventually leading to apoptosis in HepG2 cells. The results indicate a protective role of GSTP1 by enhancing GSH-conjugation, which might lead to a reduced protein modification, and suggest a second protective role by interfering with the unfolded protein response, thereby preventing apoptosis.

## Author Contributions

YZ, SdB-S, MdB, and SH conducted the experiments and performed the statistical analysis. YZ and SdB-S wrote the first draft of the manuscript. MdB, SH, JV, and JC wrote sections of the manuscript. All authors contributed to conception and design of the study and manuscript revision; read and approved the submitted version.

## Conflict of Interest Statement

The authors declare that the research was conducted in the absence of any commercial or financial relationships that could be construed as a potential conflict of interest.

## Abbreviations

AQ: amodiaquine
AQ-SG: 5′-glutathion-*S*-yl-amodiaquine
AQ-QI: amodiaquine-quinoneimine
BSO: *L*-buthionine-sulfoximine
CDNB: 1-chloro-2, 4-dinitrobenzene
CYP: cytochromes P450
DEAQ: *N*-desethylamodiaquine
DEAQ-SG: 5′-glutathion-*S*-yl-*N*-desethylamodiaquine
DEAQ-QI: *N*-desethylamodiaquine-quinoneimine
DMEM: Dulbecco’s modified Eagle medium
DMF: *N,N*-dimethylformamide
ER: endoplasmic reticulum
FBS: fetal bovine serum
GSH: glutathione
GSSG: glutathione disulfide
GST: glutathione *S*-transferase
H_2_O_2_: hydrogen peroxide
HClO_4_: perchloric acid
HRP: horseradish peroxidase
NQO1: NAD(P)H:quinone oxidoreductase 1
NAPQI: *N*-acetyl-*p*-benzoquinonimine
PBS: phosphate-buffered saline
QI: quinoneimine.
