# Supplementary material for: Glutathione S-Transferase P1 Protects Against Amodiaquine Quinoneimines-Induced Cytotoxicity but Does Not Prevent Activation of Endoplasmic Reticulum Stress in HepG2 Cells
Source: Front Pharmacol. 2018 Apr 18;9:388. doi: 10.3389/fphar.2018.00388 (PMC5915463; doi:10.3389/fphar.2018.00388)
Supplement: Supplementary file 1 [file Presentation_1.ZIP › Supplementary materials-revised.docx]

**Supplementary Materials**

**Glutathione *S*-transferase P1 protects against amodiaquine quinoneimines-induced cytotoxicity but does not prevent activation of endoplasmic reticulum stress in HepG2 cells**

Yongjie Zhang * ^# 1, 2^, Shalenie P. den Braver-Sewradj ^# 1^, Michiel W. den Braver ^1^, Steven Hiemstra ^3^, Nico P.E. Vermeulen ^1^, Bob van de Water ^3^, Jan N.M. Commandeur ^1^, J. Chris Vos ^1^

*^1^AIMMS-Division of Molecular Toxicology, Department of Chemistry & Pharmaceutical Sciences, Vrije Universiteit Amsterdam, Amsterdam, The Netherlands*

*^2^Clinical Pharmacokinetics Research Laboratory, School of Basic Medicine and Clinical Pharmacy, China Pharmaceutical University, Nanjing, China.*

*^3^Division of Drug Discovery and Safety, Leiden Academic Centre for Drug Research, Leiden University, Leiden, The Netherlands*

*** *Correspondence*

*AIMMS-Division of Molecular Toxicology, Department of Chemistry & Pharmaceutical Sciences, Vrije Universiteit Amsterdam, O|2 building, De Boelelaan 1108, 1081 HZ Amsterdam, The Netherlands*

E-mail: zhangyongjie1989@hotmail.com

*Tel.: +31 205988194*

^#^ Authors contributed equally to this manuscript


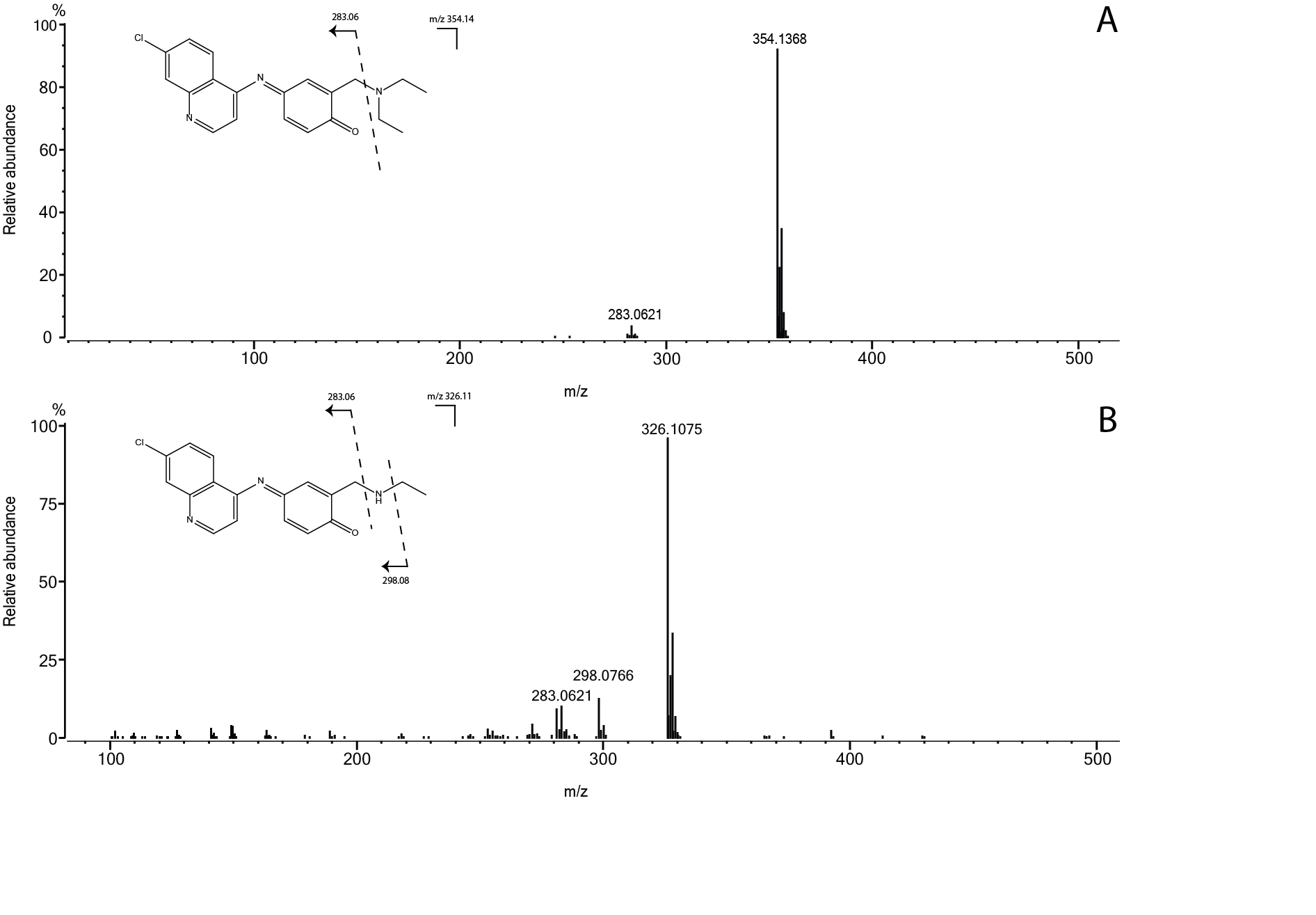


Supplementary Figure S1. Mass spectra and assignment of ion fragments of synthetic AQ-QI (A) and DEAQ-QI (B).

Supplementary Figure S2. Effect of DMF concentrations on HepG2 cell viability. HepG2 cells at density of 1×10^4^ per well were treated with BSO 24 h prior to exposure. Different concentrations of DMF were treated to cells for 2.5 h. Cell viability was measured as described in Materials and Methods section. Bars represent the mean ± SD (n=10).


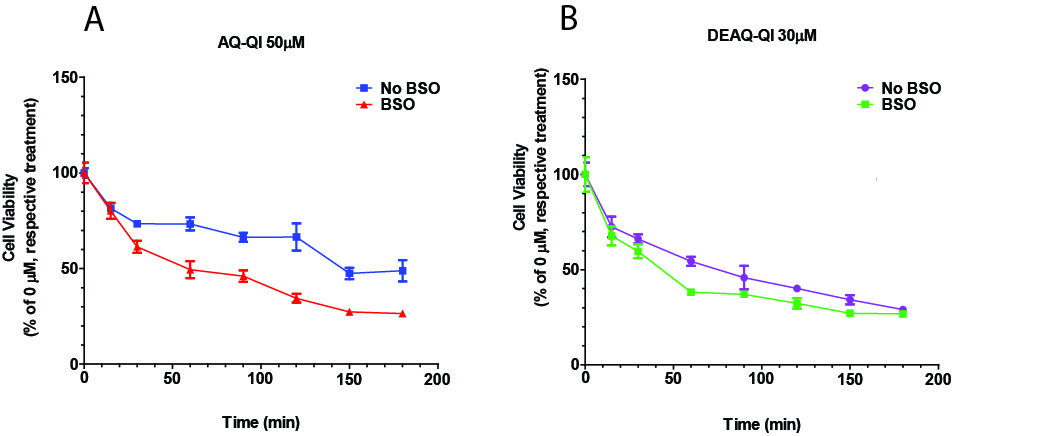


Supplementary Figure S3. Time dependency of viability of HepG2 cells upon the exposure of AQ-QI (A) and DEAQ-QI (B). Cells at density of 1×10^4^ per well were exposed to 50 µM AQ-QI (A) and 30 µM DEAQ-QI (B) for different time periods and cell viability was measured after 16 h of each exposure with resazurin reduction assay, as described in details in Materials and Methods section. Each data point represents mean ± SD (n=3).


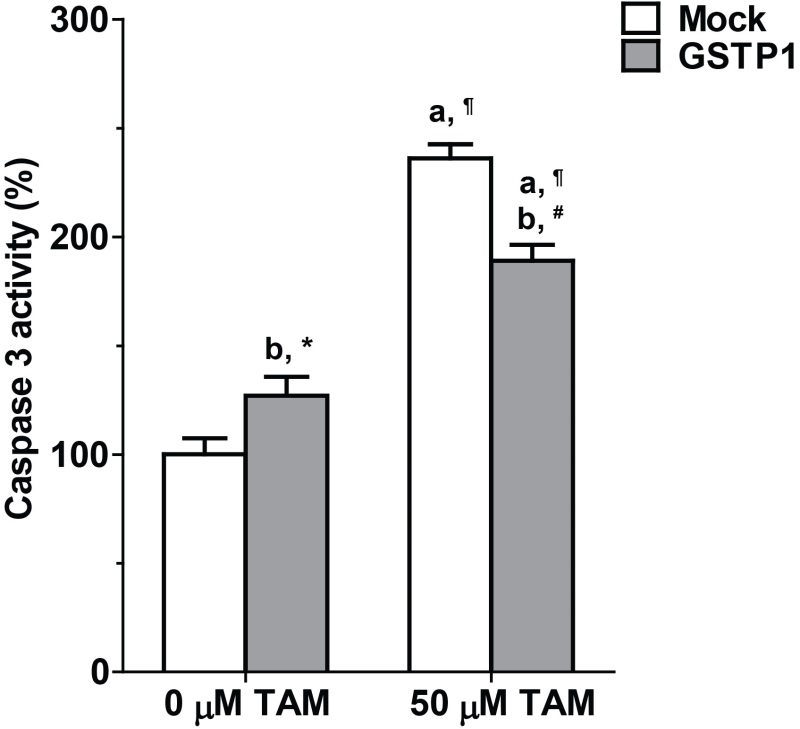


Supplementary Figure S4. Caspase 3 activity in mock- and *GSTP1*-transfected HepG2 cells following a 2.5 h exposure to 50 µM Tamoxifen (TAM) as a positive control. Caspase 3 activity was used as measure for cell death by apoptosis and is presented as percentage relative vehicle control (0.5% DMSO) in mock-transfected cells. Each bar represents the mean ± SD (n=3). Statistical significant differences with vehicle control are denoted with ‘a’ and with corresponding mock-transfected control are denoted with ‘b’, using the following *p* values: * *p* < 0.05; # *p* < 0.01; ¶ *p* < 0.001 (Student’s *t* test).


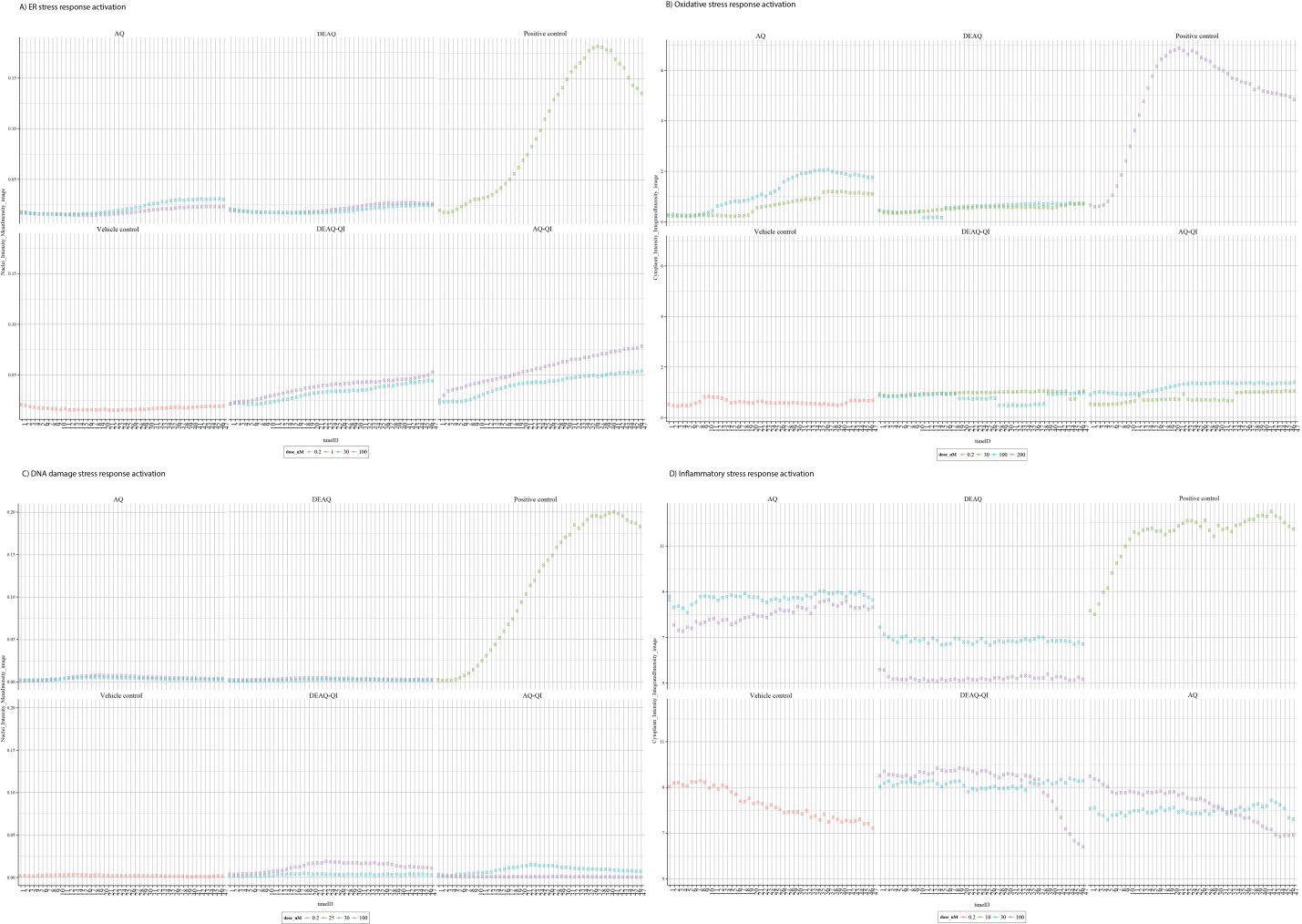


Supplementary Figure S5. Raw fluorescence signals of CHOP (panel A, ER stress response), SRXN1 (panel B, oxidative stress response), p21 (panel C, DNA damage response), and ICAM1 (panel D, inflammatory stress response) up-regulation in *mock*-transfected HepG2 cells, using high-content imaging-based BAC-GFP reporters. Signals were recorded over 48 h after exposure to AQ, DEAQ, AQ-QI, or DEAQ-QI at 30 and 100 µM, respectively. Vehicle controls are 0.2% DMF (*v/v*). Thapsigargin (1 µM), diethylmaleate (200 µM), etoposide (25 µM) and TNFα (10 µM) were included as positive controls for repectively CHOP, SRXN1, p21 and ICAM1 (Wink et al. 2014; Wink et al. 2017).
